# ASSESSING APATHY IN LONG-TERM CARE RESIDENTS WITH DEMENTIA: COMPARING SELF-EVALUATION WITH PROXY RATINGS

**DOI:** 10.1093/geroni/igz038.433

**Published:** 2019-11-08

**Authors:** Ying-Ling Jao, Kristine N Williams, Diane Berish

**Affiliations:** 1 Pennsylvania State University, University Park, Pennsylvania, United States; 2 University of Kansas Medical Center, Kansas City, Kansas, United States

## Abstract

Background: Apathy affects most individuals with dementia in long-term care. Apathy assessment is fundamental for appropriate treatment. Apathy involves subjective feelings thus individual’s self-evaluation may offer important perspectives for assessment. However, it is unclear whether self-evaluation is a valid assessment approach for this population. This study compared apathy ratings from resident self-evaluation to assessments from family, clinicians, and research staff. Methods: This pilot study enrolled 8 residents from two long-term care facilities in Pennsylvania. One family member, one certified nursing assistant (CNA), and one nurse or activity staff were also enrolled for each resident. Researchers interviewed each resident using the Apathy Evaluation Scale (AES) and rated their apathy levels. Family, CNAs, and nurses/activity staff independently rated the resident’s apathy level using the AES. Direct observations were conducted by researchers using the Person-Environment Apathy Rating (PEAR). Results: Correlation analysis revealed a discrepancy across raters in assessing apathy. While self-evaluation and family ratings where moderately positively correlated (r=0.48, p=.23), there was a moderate correlation in the opposite direction between self-evaluation and CNA ratings (r=-0.64, p=.09). Resident self-evaluation did not correlate with nurses/activity staff ratings (r=0.01, p=.99) or researcher observations (r=-0.08, p=.86). Discussion: These findings may reflect residents’ cognitive impairment and lack of insights, family and clinicians’ lack of understanding of apathy, or nurses’ and researchers’ lack of acquaintance with the resident. It remains undetermined whether self-evaluation provides valid information for apathy assessment for this population. Additional research is necessary to identify the most valid assessment approach for long-term care residents with dementia.

